# The over-estimation of distance for self-voice versus other-voice

**DOI:** 10.1038/s41598-021-04437-8

**Published:** 2022-01-10

**Authors:** Wen Wen, Yuta Okon, Atsushi Yamashita, Hajime Asama

**Affiliations:** 1 Research Into Artifacts, Center for Engineering, The University of Tokyo, 7-3-1 Hongo, Bunkyo-ku, Tokyo, 113-8656 Japan; 2 Department of Precision Engineering, The University of Tokyo, 7-3-1 Hongo, Bunkyo-ku, Tokyo, 113-8656 Japan

**Keywords:** Psychology, Human behaviour

## Abstract

Self-related stimuli are important cues for people to recognize themselves in the external world and hold a special status in our perceptual system. Self-voice plays an important role in daily social communication and is also a frequent input for self-identification. Although many studies have been conducted on the acoustic features of self-voice, no research has ever examined the spatial aspect, although the spatial perception of voice is important for humans. This study proposes a novel perspective for studying self-voice. We investigated people’s distance perception of their own voice when the voice was heard from an external position. Participants heard their own voice from one of four speakers located either 90 or 180 cm from their sitting position, either immediately after uttering a short vowel (i.e., active session) or hearing the replay of their own pronunciation (i.e., replay session). They were then asked to indicate which speaker they heard the voice from. Their voices were either pitch-shifted by ± 4 semitones (i.e., other-voice condition) or unaltered (i.e., self-voice condition). The results of spatial judgment showed that self-voice from the closer speakers was misattributed to that from the speakers further away at a significantly higher proportion than other-voice. This phenomenon was also observed when the participants remained silent and heard prerecorded voices. Additional structural equation modeling using participants’ schizotypal scores showed that the effect of self-voice on distance perception was significantly associated with the score of delusional thoughts (Peters Delusion Inventory) and distorted body image (Perceptual Aberration Scale) in the active speaking session but not in the replay session. The findings of this study provide important insights for understanding how people process self-related stimuli when there is a small distortion and how this may be linked to the risk of psychosis.

## Introduction

Self-related stimuli, such as self-names, self-face, self-body, and self-voice, hold a special status in our perceptual system^1,2^. For example, one’s own name and face attract attention^3,4^ and trigger larger amplitudes of brain activity^5,6^ compared to others’ names and faces. For another example, the recognition of self-body is also faster and more accurate than recognition of other-body^7–9^. Furthermore, pre-recorded self-generated voices generate larger event-related potential components than non-self-voices in an oddball paradigm, thereby showing that one’s own voice has greater affective salience than an unfamiliar voice^10^. Moreover, recognizing oneself as the owner of names, faces, a body, and voices may rely on a common representation network between self and others^1^.

Voices play an important role in daily social communication. Self-voice is a frequent input for self-identification. Neuroimaging studies show that listening to one’s own voice increases brain activity in the inferior frontal brain regions, which is largely similar to that for processing self-related stimuli across modalities^11–14^. However, little is known about people’s perceptual processes of self-voice. Previous studies on the perceptual processing of self-voice showed that people are less accurate in recognizing self-voice than other-voice^15^, although the former is considered highly familiar for people. Specifically, when participants were asked to judge if a voice was their own or not, they often misattributed their own voice to others^15^. Interestingly, the phenomenon of less accurate judgment for self-voice than other-voice was only observed when the participants were required to make explicit recognition judgments but absent when making implicit judgments where two voices were uttered by the same or different people^15^. Electrophysiological studies showed that self-voice triggered smaller P3a—an event-related potential reflecting the involuntary orientating of attention^16^—in an odd-ball paradigm compared to other-voices^17,18^. This indicated that fewer pre-attentional resources are involved in the processing of self-voice^18^.

Many previous studies have also focused on the acoustic features of self-voice for voice recognition, showing that the fundamental frequency and formant structures serve as key acoustic cues for self-voice identification^19^. Distortions of acoustic features using pitch-shift greatly impair people’s ability of self-recognition even when they hear the voice feedback in real-time^20^. Studies on schizophrenia have also focused on self-voice perception because of the link between self-voice recognition and auditory hallucination, which is a major feature of this disease. Patients with schizophrenia often hear voices of other people speaking to them. Psychological and psychiatric studies suggest that auditory hallucinations probably arise from the impairment in monitoring one’s own inner speech, resulting in the misattribution of one’s own inner voice as an external voice^21^. Neuroimaging studies have shown that the production of auditory hallucinations in schizophrenia is associated with increased activity in the brain regions of language^22,23^, thus, supporting the link between abnormal processing of inner speech and auditory hallucinations. Moreover, behavioral studies have shown that compared to healthy controls, patients with schizophrenia make significantly more misattributions of their own voice to others when there is an acoustic distortion in real-time feedback of their own voice^14,24–27^. Additionally, patients with left and right brain damage showed different impairments in self-voice recognition, either misattributing others’ voices to themselves or denying the ownership of self-voice^28^.

In contrast to numerous studies on the acoustic features of self-voice, little is known regarding the temporal and spatial processing of self-voice. A previous study using event-related potentials showed that delayed self-voice feedback elicited significantly larger neural responses than pitch-shifted self-voice feedback, indicating that the neural processing of auditory self-voice feedback is highly sensitive to its temporal features^29^. A recent preprint investigated the time perception of non-altered and acoustic distorted voice feedback, showing that the perceived interval between a person’s speech and non-altered voice feedback was significantly shorter than that between the former and acoustic distorted voice feedback^30^. This phenomenon was explained by a strong sense of agency, which is considered to compress the time perception between voluntary actions and sensory feedback^31^ for non-altered self-voice^30^.

In contrast to studies on perceptual processing of acoustic and temporal features of self-voice, surprisingly, there is no study on the spatial perception of self-voice. We usually hear our own voices from our own location when we speak. On some occasions, such as echoes in online chatting, replays of recordings, and echoes from a mountain, we hear our own voices from a different location. People often feel that their own voices from other sources sound different from their known self-voices. This well-known phenomenon is usually attributed to a lack of bone conduction^32–34^. However, the spatial discrepancy in self-voice may be another possible cause for this “weird” feeling. Nevertheless, the spatial perception of self-voice when hearing it from an external source remains unknown. Additionally, previous studies reported that patients with schizophrenia who experienced auditory hallucinations heard voices both inside and outside their heads^35^. Moreover, a neuroimaging study reported that the spatial location of auditory hallucinations is associated with the right temporoparietal junction—a region of spatial localization in auditory processing^36^. Spatial localization of self-voice may be a key feature in the assessment of psychoses associated with auditory hallucinations. Therefore, the questions arise: When there is a spatial distortion of self-voice, how would people process such distortion? Would the processing of spatial distortion in self-voice be linked with schizotypal personality? The previous studies found that healthy participants often misattribute the self-voice to others when they make explicit judgments of ownership^15^, and patients with schizophrenia misattribute self-voice to others more often than healthy people^14,24–27^, probably because people tend to overestimate the acoustic distortion in voice feedback. We also predict that in the case of spatial distortion in self-voice, people with stronger schizotypal personality may tend to overestimate the spatial distortion.

In the present study, participants heard either a non-altered self-voice (i.e., the self-voice condition) or an acoustically distorted self-voice stimulus (i.e., the other-voice condition; the self-voice was pitch-shifted upward or downward by four semitones in this condition) from one out of four speakers that were located either 90 or 180 cm away from their sitting position. The distances were selected based on our pilot investigation, which aimed to find suitable distances to avoid both ceiling and floor effects. The voice stimulus was present immediately after articulation or was a replay of prerecorded voices presented without articulation. The participants indicated the speaker from which they heard the voice stimulus. The accuracy of spatial localization was compared between self-and other-voice conditions for each speaker. An additional questionnaire of schizotypy was administered to a subset of the participants for structural equation modeling (SEM) to examine the relationship between individual schizotypal personality traits and behavioral performance in the spatial perception of self-voice.

## Material and methods

### Participants

Thirty-one university students (mean age = 22.5 years, *SD* = 2.0 years, 11 females) were recruited using a university-wide social media advertisement. All participants had normal auditory acuity and no psychiatric disorders. The experiment was conducted with the approval of the ethics committee of the Faculty of Engineering at the University of Tokyo, Japan, and performed in accordance with relevant guidelines and regulations. Written informed consent was obtained from all participants prior to the experiments, and participants received financial compensation for their participation.

Based on our pilot experiments, we selected the experimental parameters (e.g., distance and number of speakers) to achieve an average accuracy of approximately 70% and set an exclusion criterion based on the performance of a baseline task before the actual sessions to exclude participants who performed at chance levels. Specifically, participants whose distance judgment accuracy of the sound source was equal to or below 50% (i.e., the chance level was 50% because there were only two types of distances) in a passive hearing session before the actual task were removed from the data analyses. Seven participants were excluded based on this criterion, resulting in a sample size of 24 for the behavioral task. We could not perform a power calculation because no previous studies, to our knowledge, had investigated the influence of self-voice on distance perception. We conducted a post-hoc power analysis and showed that the sample size was sufficient to examine the effect of self-voice on spatial judgment.

In addition to the main behavioral task, we conducted an online questionnaire survey using three scales of schizotypy. Twenty-one participants who also took part in the behavioral task responded to the questionnaire. We excluded participants whose scores exceeded ± 3 *SD* of the group average because rare deviates would influence the results of multivariable analysis based on covariance, diminishing the common tendency in the population of interest. Two participants were excluded from this analysis, resulting in a sample size of 19 for multivariable analysis. This study’s design and hypotheses were preregistered.

### Behavioral task and procedure

The auditory localization task was programmed using MATLAB with Audio Toolbox (R2019a, Mathworks, US) and Psychtoolbox-3^37,38^. The visual instructions were presented on a 27-in. LED monitor. Four sets of stereo speakers (MM-SPL2N2, Sanwa, Japan) were used to present the auditory stimuli. The left and right speakers of each set were attached together as a single unit. The volume of all speakers was adjusted to be identical at a comfortable level using a volume indicator. A four-channel headphone amplifier (HA400, Ammoon, China) was used to switch the output of the speakers. A clip microphone (HS-MC06BK, Elecom, Japan), fixed to participants’ collars, was used to record their voices.

**Figure 1:**
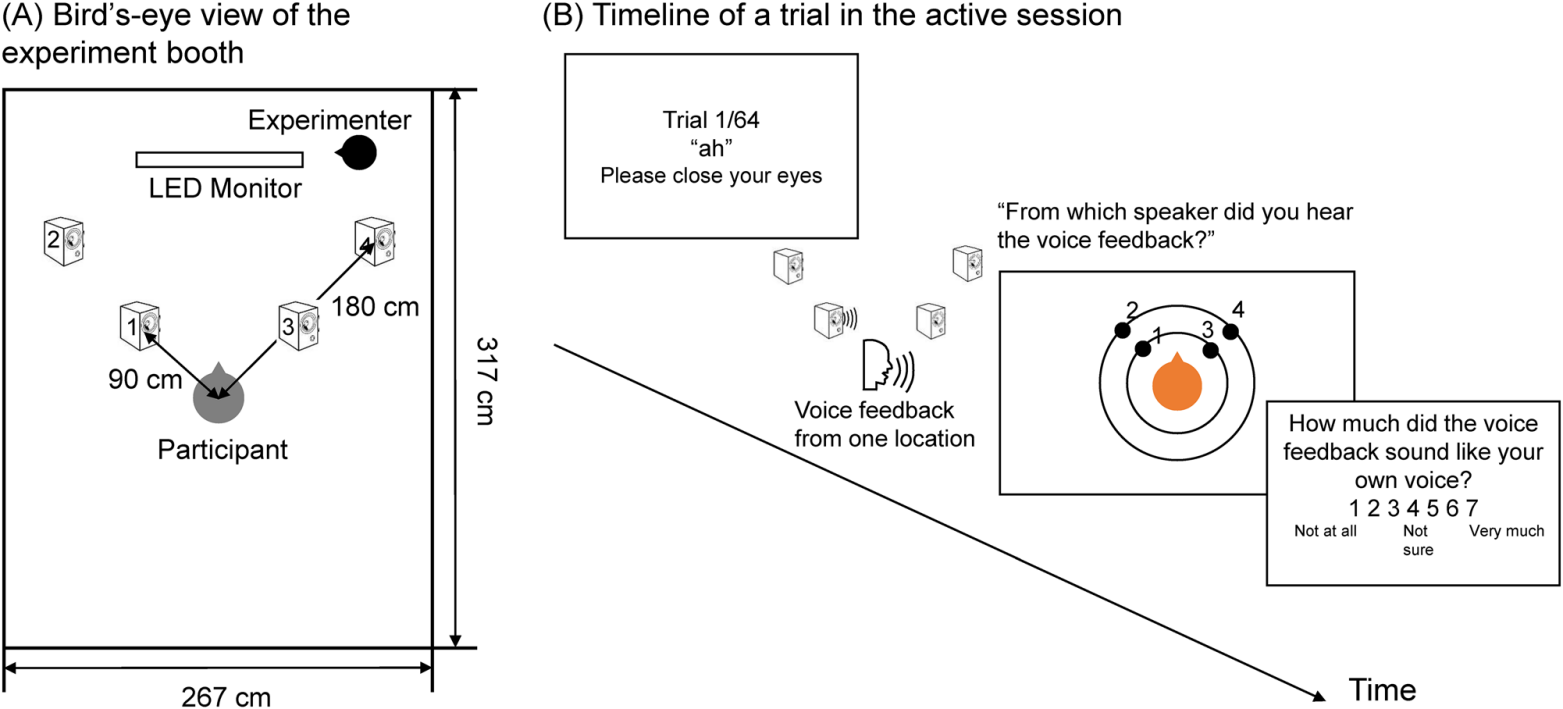
Setup of the experiment devices (**A**), and the timeline of a trial in the active session (**B**).

Behavioral experiments were conducted individually in a sound-insulated booth. Figure 1A shows a bird’s-eye view of the booth, and Fig. 1B shows the timeline of the trial during the active session. The four speakers were allocated 45º left front or right front, 90 or 180 cm away from the center of the participants’ sitting position. Labels–1–4 were attached to the top of each speaker, and the numbers were also shown on the screen when participants’ responses to the sound source were required. The auditory localization task contained three sessions: a passive session, an active session, and a replay session. After being introduced to the task, the participants first performed a passive session. This session was designed to measure the baseline performance of the auditory localization task and exclude participants who performed at the chance level. In each trial, participants heard a prerecorded unknown voice pronouncing “ah” (i.e., the first vowel sound in the Japanese syllabary) from one of the four speakers with their eyes closed. One of the two prerecorded voice stimuli of either female or male tone was used according to the participants’ gender. Three seconds after the onset of the voice stimulus, participants opened their eyes according to the experimenter’s verbal instruction. The participants then verbally identified the number of the speaker they thought carried the voice stimulus. A map showing the speaker numbers was presented on the screen. The passive session did not apply any pitch-shifting to voice stimuli. This session contained 8 practice trials, followed by 20 actual trials, with an equal number of trials for each speaker; the trial order was randomized. Participants were given feedback on the correct sound source in the practice trials but not in the actual trials of the passive session.

**Figure 2:**
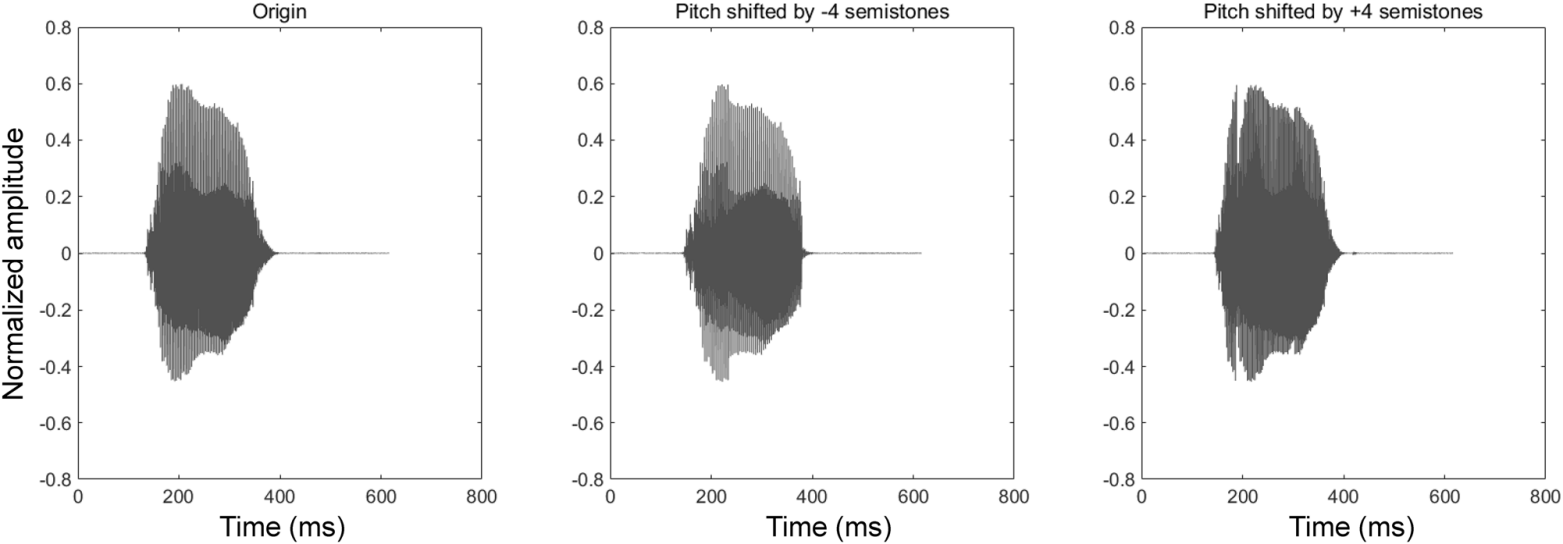
Examples of a voice speaking “ah” with and without pitch-shifting. Y-axes are normalized amplitude ranging from − 1 to + 1, corresponding to the minimum and maximal voltage that can be produced by the line driver, respectively.

Following the passive session, the participants engaged in the active session. In each trial of the active session, participants uttered the Japanese vowel “ah” and heard the voice feedback from speakers with their eyes closed. The voice stimulus was processed in real-time using the Pitch Shifter of MATLAB Audio Toolbox, and feedback was provided to the participants via one of the four speakers. The voice stimuli were pitch-shifted by 0, + 4, or − 4 semitones. Figure 2 shows examples of the original voice reading “ah” and pitch-shifted voices. The Pitch Shifter did not affect the intensity of the voices, which is critical for the distance judgment of sound^39,40^. Owing to the processing time of the device, there was a delay of approximately 300 ms between articulation and voice feedback. Participants heard the voice feedback 300 ms *after* the onset of their articulation. This avoided the overlap between the speaking and the voice feedback from the speakers. Participants were told to speak in a constant tone and normal talking volume in all the trials. The time window for speaking and voice feedback was 3 s. Thereafter, participants opened their eyes and verbally identified the number of the speaker they thought carried the voice feedback. Participants then verbally rated how much they felt that the voice sounded like their own voice using a 7-point Likert scale from 1 to 7 (1 = not at all, 4 = not sure, 7 = very much). There were 8 trials without pitch-shifting, 4 with a pitch-shift of + 4 semitones, and 4 with a pitch-shift of − 4 semitones for each speaker, resulting in 64 trials in total in this session. The trial order was randomized. The participants performed eight practice trials prior to the actual trials. There was no feedback regarding the accuracy of responses in either the practice or actual trials.

Finally, the participants performed the replay session. Participants’ voices pronouncing “ah” were recorded at the beginning of each experiment and were the recorded voices used in this session. The timeline of the replay session was identical to the active session, except that participants did not speak but only heard the replay of their own voices with their eyes closed. The number of conditions and trials were the same as in the active session. This replay session was designed to examine whether the action of speaking is necessary for any potential effect of self-voice on spatial localization. The behavioral experiment took approximately 60 min per participant, including document filling, introduction, practice, and actual tasks.

### Schizotypy questionnaire

The schizotypy questionnaire was administered 1–30 days after the behavioral task. Three scales were included based on a previous study on the relationship between schizotypy scores and implicit sense of agency^41^: Peters Delusion Inventory (PDI)^42^, the Magical Ideation Scale (MIS)^43^, and the Perceptual Aberration Scale (PAS)^44^. The PDI contains 21 items, measuring delusional ideation by asking people to rate their experiences and thoughts, such as “Do you ever feel as if people are reading your mind?”^42^. The MIS contains 30 items, assessing beliefs that are inconsistent with the cultural standard by asking people to provide true/false responses to statements such as: “Some people can make me aware of them just by thinking about me”^43^. The PAS contains 35 items, assessing distortions in the perceptual experience of one’s body and surrounding spaces by asking people to provide true/false responses to items such as: “I have felt that something outside my body was a part of my body”^44^. These three scales were translated into Japanese by a native Japanese speaker, and both Japanese and English expressions were presented to the participants. All participants were native Japanese speakers with English as their second language. The questionnaire was administered using an online Google Form. Participants reported to the experimenter before and after answering the questionnaire via email. The questionnaire took each participant 20–30 min to complete. All the stimuli and codes will be available upon request to the corresponding author.

### Data analysis

Task performance in the passive session was used to exclude poor performers in the auditory localization task and was not included in the statistical analyses. In addition, task performance could not simply be compared between the passive session and the other two sessions because the auditory stimuli had very different auditory intensities, and the voice intensity greatly affected people’s distance perception^39,40^.

First, we compared the self-voice likelihood ratings among voice distortion conditions to check whether the manipulation of voice acoustic features to produce self versus other perceptions was successful. A 3 × 2 (pitch-shifting: 0, + 4, vs. − 4 semitones × session: active vs. replay) repeated-measures ANOVA was conducted for the rating scores. Post-hoc comparisons, using Bonferroni corrections, were conducted for significance(s). We predicted a significant difference in the rating scores between the non-altered condition (i.e., 0 semitones) and the other two distorted conditions (i.e., + 4 and − 4 semitones), but no significant difference between the two distorted conditions. After confirming this point, we categorized the three voice distortion conditions into two voice-type conditions: self-voice and other-voice. The self-voice condition refers to one in which a non-altered self-voice is presented. The other-voice condition contained two pitch-shifted conditions (by + 4 and − 4 semitones).

For spatial judgment, we focused on auditory distance perception. The direction judgment (i.e., left front vs. right front) was highly accurate (> 99%). Trials with incorrect direction judgments were removed from the analyses. An angular transformation was applied to normalize the distribution of the accuracy results. The normality of the data sets after angular transformation was confirmed using the Shapiro–Wilk test. We conducted 2 × 2 (type of voice: self vs. other × session: active vs. replay) repeated-measures ANOVAs for the judgment accuracy of each distance condition (i.e., when the speakers are closer and further away). All ANOVAs were conducted using IBM SPSS Statistics 23.

Finally, the collected individual schizotypal scores and behavioral performances were pooled into a structural equation model. The model contained five variables, including three schizotypy scores and two behavioral indices. The two behavioral indices were the differences in accuracy between the self-and other-voice conditions in the active and replay sessions, respectively. SEM was conducted using IBM SPSS Amos 22. All the datasets will be available upon request to the corresponding author.

## Results

### Self-voice likelihood rating

**Figure 3:**
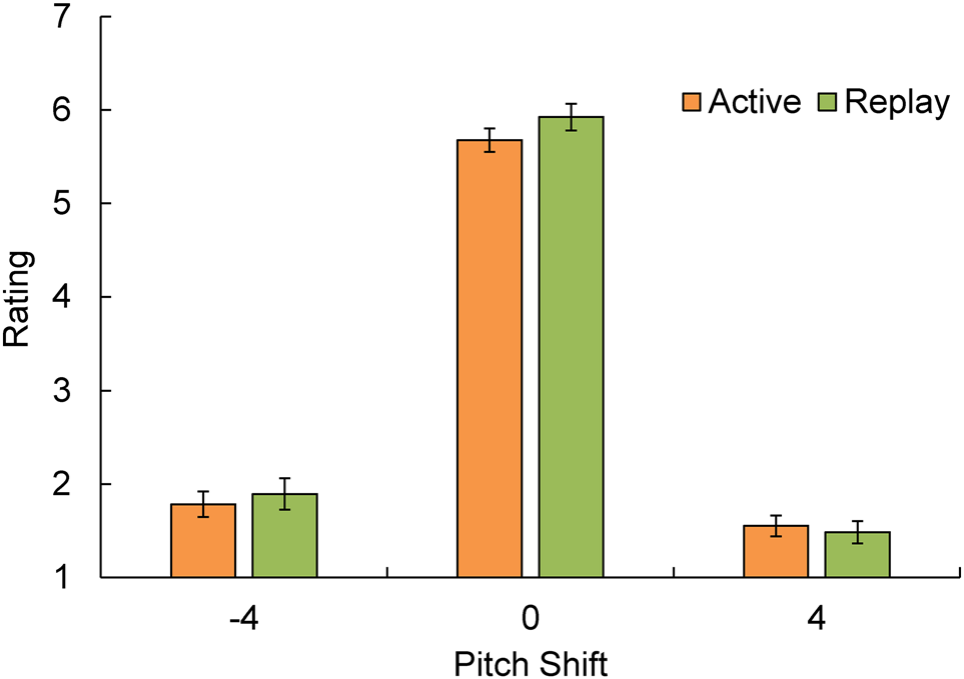
Rating score in each voice condition of the active and replay sessions. Error bars represent standard errors.

Figure 3 shows the self-voice likelihood rating scores for each voice distortion condition in the active and replay sessions. A 3 × 2 (pitch-shift: − 4, 0, vs + 4 semitones × session: active vs. replay) ANOVA revealed a significant main effect of pitch-shift (*F*(1, 23) = 286.59, *p* < 0.001, partial η^2^ = 0.926), and a significant main effect of session (*F*(1, 23) = 5.52, *p* = 0.028, partial η^2^ = 0.194). The interaction between pitch-shift and session was also significant (*F*(2, 46) = 3.51, *p* = 0.038, partial η^2^ = 0.132). The main effect of the session showed that the ratings in the replay session were higher than those in the active session, probably because the participants were able to concentrate on the acoustic features of the voice feedback when they did not need to speak in the replay session as compared to the active session. The significant interaction was probably because the participants tended to rate higher in the replay session for the conditions of 0 and − 4 semitones but not for the condition of + 4 semitones than the active session. However, this was not our study’s primary focus. Six post-hoc comparisons (3 comparisons: 0 semitones vs. + 4 semitones, 0 semitones vs. − 4 semitones, + 4 semitones vs − 4 semitones × 2 sessions) were conducted using the Bonferroni correction (i.e., the significance level was set to 0.05/6 = 0.0083). The results confirmed our predictions that non-altered voice feedback was rated as sounding like their own voices, while pitch-shifted voices were rated as not sounding like their own voices. The differences between the non-altered condition and the two distorted conditions were significant in both sessions (for the active session, 0 semitones vs. − 4 semitones, M = 5.68 vs. 1.78, SD = 0.64 vs. 0.69, *t*(23) = 17.35, *p* < 0.001; 0 semitones vs. + 4 semitones, M = 5.68 vs. 1.55, SD = 0.64 vs. 0.56, *t*(23) = 22.37, *p* < 0.001; for the replay session, 0 semitones vs. − 4 semitones, M = 5.92 vs. 1.89, SD = 0.71 vs. 0.84, *t*(23) = 14.35 *p* < 0.001; 0 semitones vs. + 4 semitones, M = 5.68 vs. 1.48, SD = 0.64 vs. 0.60, *t*(23) = 20.88, *p* < 0.001). The differences between the two distorted conditions were non-significant in both sessions (for the active session: − 4 semitones vs. + 4 semitones, M = 1.78 vs. 1.55, SD = 0.69 vs. 0.56, *t*(23) = 1.71, *p* = 0.101; for the replay session: − 4 semitones vs. + 4 semitones, M = 1.89 vs. 1.48, SD = 0.84 vs. 0.60, *t*(23) = 2.26, *p* = 0.034). In the rest of the analyses, the non-altered condition was named the self-voice condition, and the two distorted conditions were mixed and named the other-voice condition.

### Auditory spatial perception accuracy

**Figure 4:**
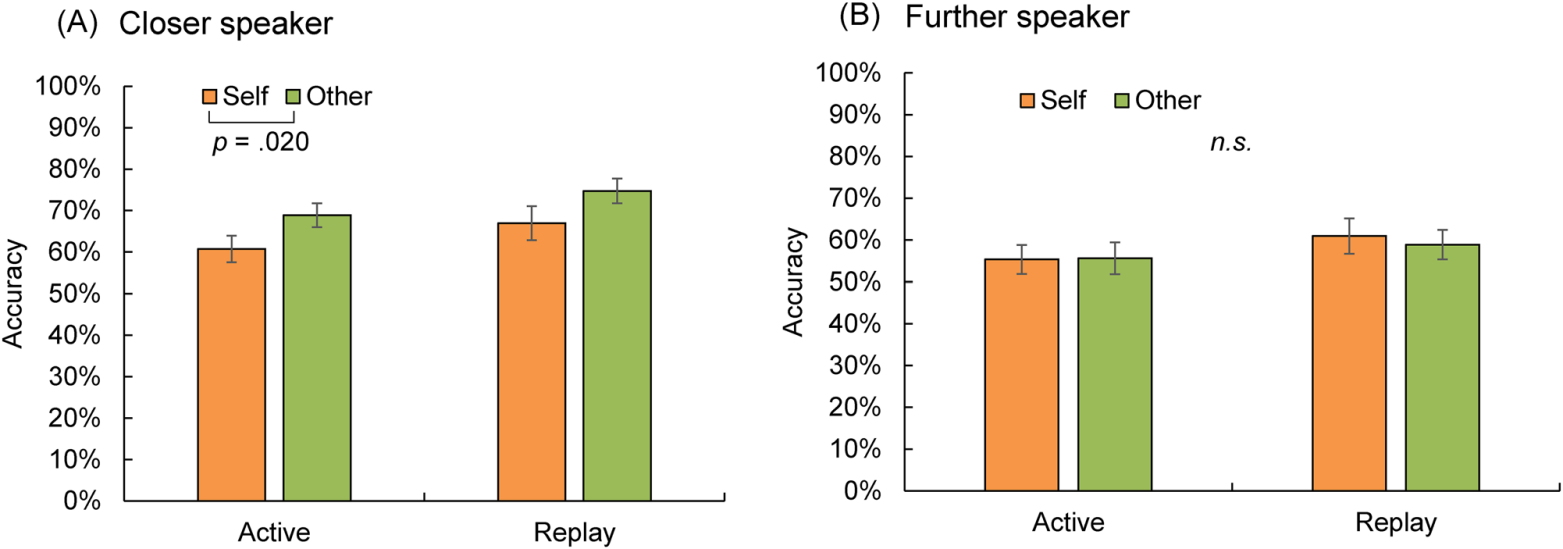
Distance judgment accuracy for the voice stimuli that were presented from the closer speakers (i.e., 90 cm) and those further away (i.e., 180 cm). The effect of type of voice (self vs. other) was significant for the closer speakers but non-significant for the speakers further away. This indicated that participants tended to overestimate the distance for the self-voice instead of just making a poorer spatial judgment for the self-voice compared to that for the other-voice. Error bars represent standard errors.

Figure 4A,B show the accuracy of spatial judgment for the speakers closer and further away, respectively. We did not include the two presented distances as an independent variable in the statistical analyses because we wanted to examine whether participants perceived the sound source to be further or closer than their actual location instead of the judgment accuracy. Trials with incorrect direction judgment (i.e., left front vs. right front) were excluded from the analyses (0.5% of trials); thus, the performance reflected auditory distance perception. Regarding the voice stimuli that were presented from the closer speakers, the 2 × 2 (type of voice: self vs. other × session: active vs. replay) repeated-measures ANOVA revealed a significant main effect of the type of voice (*F*(1, 23) = 6.25, *p* = 0.020, partial η^2^ = 0.214). The accuracy in the self-voice condition (i.e., non-altered voice) was significantly lower than that in the other-voice condition (i.e., pitch-shifted voice). The main effects of the session and interaction were not significant (*F*(1, 23) = 3.92, *p* = 0.060, partial η^2^ = 0.146; *F*(1, 23) = 0.02, *p* = 0.883, partial η^2^ = 0.001, respectively). In short, participants tended to overestimate the spatial distortion when they heard their own voice from a location away from themselves, attributing the sound source to an even further one compared to the condition when they heard someone else’s voice. However, one may argue that this result reflected poorer spatial judgment for self-voices than other-voices. This can be tested by the distance judgment in the conditions when the voices were presented by speakers further away.

The ANOVA on the judgment accuracy when the voice stimuli were presented from the speakers further away did not find any significant main effect or significant interaction (the main effect of type of voice: *F*(1, 23) = 0.064, *p* = 0.802, partial η^2^ = 0.003; the main effect of session: *F*(1, 23) = 2.23, *p* = 0.149, partial η^2^ = 0.088; interaction: *F*(1, 23) = 0.251, *p* = 0.621, partial η^2^ = 0.011). This result supports the overestimation account for the self-voice feedback. Specifically, self-voices played by speakers 1 and 3 were more often misattributed to speakers 2 and 4 than other-voices (see Fig. 1 for the map of speakers). This result can be either explained as an overestimation of distance or a larger misattribution of sound source for self-voice than other-voice. In the first case, we should not find any difference in the spatial judgment between self-voice and other-voice when the voices were played by speakers 2 and 4 because even if participants felt that the voice sounded further than its actual location, there were no further options for responses than speakers 2 and 4. However, in the second case, we should find a similar effect of self-voice when the voices were played by speakers 2 and 4 as when played by speakers 1 and 3. The results confirmed the first case, that is, the overestimation account. In addition, we included three distance conditions in our pilot experiments, but found that participants’ task performance was at chance even in the passive listening condition. The two distance conditions used were the simplest and met the minimum requirement to examine our research question.

Finally, we performed a post-hoc power analysis to check whether our sample size (n = 24) was sufficient to examine the main effect of voice type on the spatial judgment for the further speakers. Because the accuracy for the further speaker was poorer than the closer speaker, we reduced the effect size of the main effect of voice type from the closer speaker (partial η^2^ = 0.214) according to the ratio of judgment accuracy (mean accuracy; closer speaker = 68.0%, further speaker = 57.4%). The power analysis showed that our sample provided a power of 0.97, indicating that the lack of significance for the further speaker was not due to the sample size.

### Structural equation modeling using individual schizotypy scores

**Figure 5:**
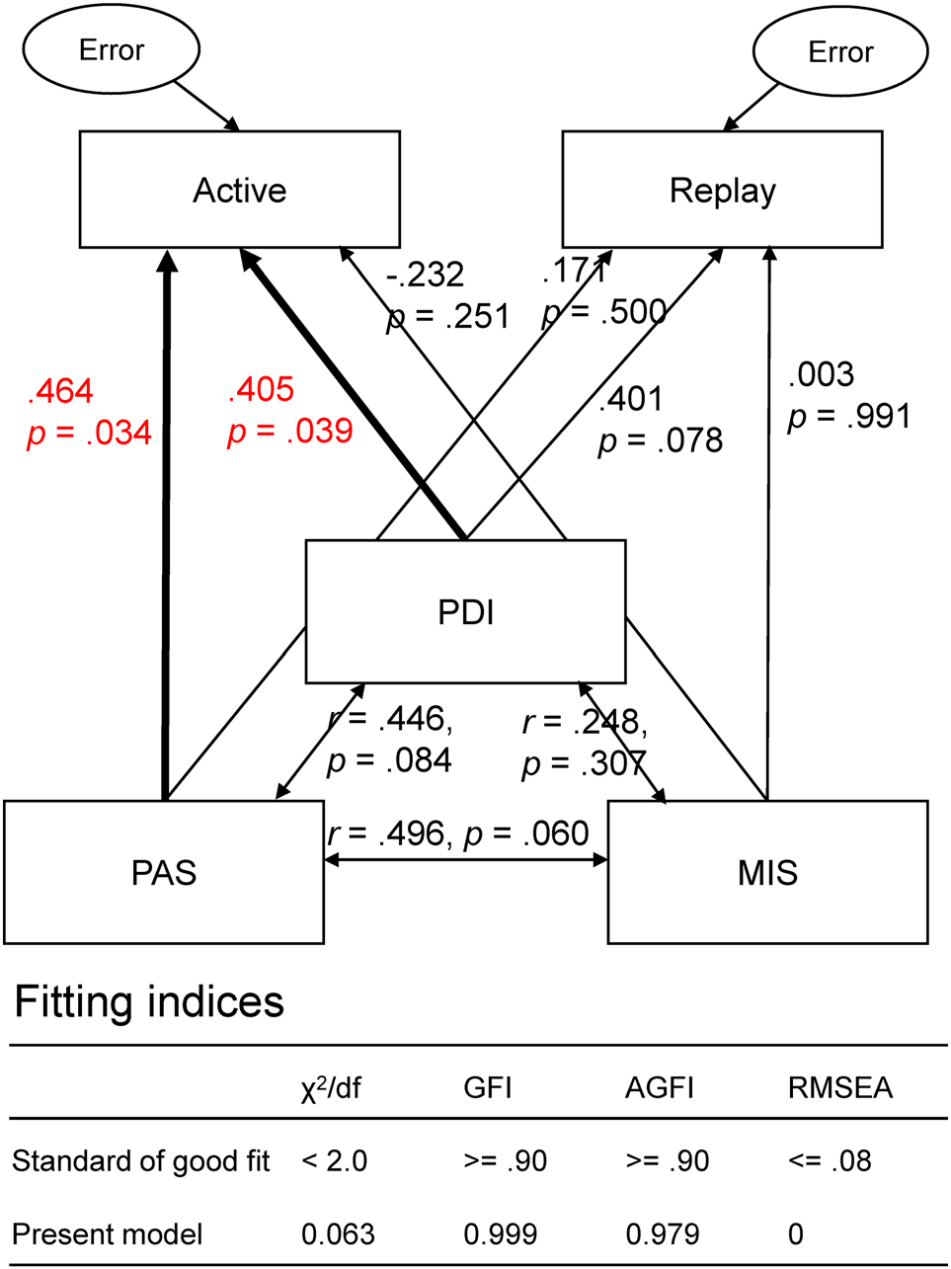
Standardized coefficients and significances of each path, and the fitting indices of the SEM. One-way arrows represent causal assumptions, and two-way arrows represent correlations. Significant causal paths were marked with bold arrows. The model fit the data set well.

After collecting participants’ schizotypy scores using online questionnaires, we conducted an SEM to examine whether the overestimation of auditory distance for the self-voice is associated with individual schizotypal personality traits. Figure 5 shows the model used for the SEM. The two effects of self-voice in the active and replay sessions were calculated by subtracting the judgment accuracy of the self-voice from that of the other-voice in the condition of closer speakers for each session (i.e., the differences between the orange and green bars in Fig. 4A). The SEM revealed significant influences of PDI and PAS scores on the effect of self-voice in the active session (standardized coefficient = 0.405 and 0.464, respectively, *p*s < 0.05). However, surprisingly, although the effect of self-voice in the replay session was also significant and comparable with that in the active session, its variance between individuals could not be explained by the schizotypy scores.

## Discussion

This study is the first to compare auditory distance perception between self-voice and other-voice. Participants heard voice feedback from one of four speakers that were located either 90 cm or 180 cm away from them after speaking a short vowel “ah.” The voice feedback was either non-altered or pitch-shifted by + 4 or − 4 semitones. Subjective ratings confirmed that pitch-shifted voices sounded like others’ voices. The results of spatial judgment showed that the distance of self-voice was significantly overestimated compared to that of other-voice. This phenomenon was also observed when the participants heard replayed self- and other-voices without speaking. Furthermore, the SEM using individual schizotypy scores showed that the effect of self-voice on distance perception was significantly associated with the score of delusional ideation (PDI) and distortions in the perceptual experience of one’s body and surrounding spaces (PAS) in the active speaking condition but not in the replay condition. Our findings provide novel knowledge for people’s spatial perception of their own voice when the self-voice is spatially distorted (i.e., from a different location than their head) and also highlight the important link between this phenomenon and schizotypal personality traits.

Previous research on self-voice has mainly focused on the acoustic features of voices. Many studies have reported that people can still identify their own voice in the condition of real-time voice feedback better than chance, even when the acoustic features of the self-voice are distorted, but there are often misattributions^19,45^. Interestingly, two recent studies showed that people are actually less accurate in making explicit ownership judgment for self-voice than other-voice^15,28^, although the former is considered more familiar. Specifically, self-voices were often misattributed to others^15^, especially in patients with right hemisphere damage^28^ and patients with schizophrenia^14,24–27^. There is a possibility that people may have over-estimated small acoustic distortions in the self-voice and had difficulties in attributing the self-voice stimuli to themselves.

However, temporal and spatial distortions do not influence the recognition of self-voice if the acoustic features are not altered. However, temporal distortions can trigger large neural responses associated with error detection^29^, indicating that the perceptual processing of self-voice is highly sensitive to temporal features. In contrast, the effect of spatial distortions on auditory perception has rarely been investigated. Our results show that spatial distortion of the self-voice is overestimated compared to that of the other-voice. People usually hear their own voices from their own locations; thus, hearing a self-voice from an external source causes a weird feeling and is usually disliked^34^. This well-known phenomenon is usually explained by the lack of bone conduction when hearing the voice from an external source^32–34^. However, a possibility may coexist: self-voice from an external source contains a spatial distortion and therefore triggers error detections for self-related stimuli in our perceptual system, similar to the case of delayed self-voice^29^. The “saliency” of a spatially distorted self-voice may be a reason for the overestimation of this spatial discrepancy compared to that in the case of other-voice.

Furthermore, the SEM revealed interesting and surprising findings regarding the link between schizotypal personality traits and distance perception of the self-voice. The results showed that PDI and PAS scores were significantly linked to the effect of self-voice on distance perception. However, this link was only found for the active session, not for the replay session. This indicates that the sensory prediction of self-voice may be an important factor for the effect of self-voice on distance perception, in addition to the saliency of self-voice. Previous studies have shown that patients with schizophrenia have more misattributions of acoustically distorted self-voice to others than healthy controls^24–26,46^. It is widely accepted that auditory hallucinations in patients with schizophrenia may be a result of misattribution of inner speech to external sources^21^, probably because the brain produces distortions (i.e., inaccurate signals) in patients with schizophrenia^47–50^. In other words, patients with schizophrenia may overestimate the “errors” when there is a small distortion (for some reason) in self-voice, potentially leading to misattributions of the self-voice to others. The distance perception of an artificially created spatial distortion may be a useful tool to examine how the human perception system processes such distortions and how perceptual processes may change in the case of schizophrenia.

## Conclusions

The present study reported a novel phenomenon wherein people overestimate the distance of the external sound source when they hear their own voices compared with when they hear the voices of others. This effect of self-voice on the spatial judgment was significantly associated with individual schizotypy scores when people received voice feedback immediately after speaking, but not when people passively heard replays of their voice. However, there are still leaves many unanswered questions. Why is the spatial distortion of the self-voice overestimated? The saliency of self-voice and sensory predictions may play a role, but these hypotheses remain untested. How do patients with schizophrenia actually process such spatially distorted self-voices? Further studies are, therefore, required. Nevertheless, the findings of this study provide important knowledge for understanding how people process the spatial “distortions” of their own voices and how this may be related to the risks and symptoms of psychosis.
